# SriniVas Sadda, Andrew Schachat, Charles Wilkinson, David Hinton, Peter Wiedemann, K. Bailey Freund, David Sarraf (eds): Ryanʼs Retina, 3 Volume Set 7th Edition, Elsevier, 2022 (eBook ISBN: 9780323722148; Hardcover ISBN: 9780323722131)

**DOI:** 10.1007/s00417-022-05874-5

**Published:** 2022-11-04

**Authors:** Bernd Kirchhof

**Affiliations:** University of Cologne, Faculty of Medicine and University Hospital Cologne, Department of Ophthalmology, Cologne, Germany

*Ryan’s Retina* edited by Steven Ryan is a successful example of multilateralism. This is the 7th and latest edition of Ryan’s standard work “Retina” from Elsevier Publishers and is now available in bookstores.

Anyone who thinks of ophthalmology as a minor matter will be enlightened by the three volumes on the retina alone. Srinvas Sadda, who succeeded the late Steve Ryan as head of Doheny’s Eye Foundation, has also accepted the editorship of “Retina.” This continues Steve Ryan’s vision of global openness, curiosity, and nurturing.

Authors and co-editors from America, Europe, Asia, Australia, and China have contributed to this current edition, which should be read by ophthalmic residents, in order to assist in giving them an overview. “Retina” is also suitable for working in basic research on the retina. However, most often, practicing ophthalmologists will use it as a reference. The main benefit from the focus of the opinion leaders in our field, for example, is when weighing up competing therapy methods. Ryan’s Retina renews itself with each edition.

Finally, artificial intelligence and the problem of segmentation in automated imaging are considered. The practicing retinologist is regularly confronted with symptoms from border areas, not only with internal medicine but also with neuro-ophthalmological differential diagnoses. In an overlapping area, symptoms can originate not only from the retina but also from superordinate central nervous structures. I would recommend a future edition to include a chapter that leads from symptom to diagnosis, such as phosphenes, glare, flare, palinopsia, and visual snow.

Also, in this 7th edition, *Ryan’s Retina* makes it easier for residents and practicing ophthalmologists to work on patients and for basic researchers to familiarize themselves with retina. We can only thank the authors, the editor in chief, and the co-editors for their self-sacrificing work. I will certainly use the digital version of *Ryan’s Retina* more often, due to access from anywhere via PC/laptop.

